# Towards uncovering the roles of switchgrass peroxidases in plant processes

**DOI:** 10.3389/fpls.2013.00202

**Published:** 2013-06-19

**Authors:** Aaron J. Saathoff, Teresa Donze, Nathan A. Palmer, Jeff Bradshaw, Tiffany Heng-Moss, Paul Twigg, Christian M. Tobias, Mark Lagrimini, Gautam Sarath

**Affiliations:** ^1^Grain, Forage and Bioenergy Research Unit, Agricultural Research Service, United States Department of Agriculture, University of NebraskaLincoln, NE, USA; ^2^Department of Agronomy and Horticulture, University of Nebraska at LincolnLincoln, NE, USA; ^3^Department of Entomology, University of Nebraska at LincolnLincoln, NE, USA; ^4^Biology Department, University of Nebraska at KearneyKearney, NE, USA; ^5^Genomics and Gene Discovery Research Unit, Agricultural Research Service, United States Department of AgricultureAlbany, CA, USA

**Keywords:** switchgrass, peroxidases, lignin, Hemiptera, biotic stress, ROS

## Abstract

Herbaceous perennial plants selected as potential biofuel feedstocks had been understudied at the genomic and functional genomic levels. Recent investments, primarily by the U.S. Department of Energy, have led to the development of a number of molecular resources for bioenergy grasses, such as the partially annotated genome for switchgrass (*Panicum virgatum* L.), and some related diploid species. In its current version, the switchgrass genome contains 65,878 gene models arising from the A and B genomes of this tetraploid grass. The availability of these gene sequences provides a framework to exploit transcriptomic data obtained from next-generation sequencing platforms to address questions of biological importance. One such question pertains to discovery of genes and proteins important for biotic and abiotic stress responses, and how these components might affect biomass quality and stress response in plants engineered for a specific end purpose. It can be expected that production of switchgrass on marginal lands will expose plants to diverse stresses, including herbivory by insects. Class III plant peroxidases have been implicated in many developmental responses such as lignification and in the adaptive responses of plants to insect feeding. Here, we have analyzed the class III peroxidases encoded by the switchgrass genome, and have mined available transcriptomic datasets to develop a first understanding of the expression profiles of the class III peroxidases in different plant tissues. Lastly, we have identified switchgrass peroxidases that appear to be orthologs of enzymes shown to play key roles in lignification and plant defense responses to hemipterans.

## INTRODUCTION

Perennial warm-season grasses such as switchgrass (*Panicum virgatum* L.), miscanthus (*Miscanthus* × *giganteus*) and giant reed grass (*Arundo donax* L.) are expected to become major sources of renewable biomass for the biofuel sector (Lewandowski et al., 2003; Vogel et al., 2011; Kering et al., 2012). Switchgrass is a focus bioenergy crop for the central regions of the US and elsewhere based on its high yield potential and other useful characteristics (Vogel et al., 2011). Switchgrass can be sustainably grown as a biofuel crop on marginal croplands with limited inputs (Schmer et al., 2006, 2008; Vogel et al., 2010). Biomass yields can be variable, but high yields are possible. Switchgrass retains a high level of genotypic and phenotypic plasticity that can be exploited for agronomic improvements (Missaoui et al., 2006; Bouton, 2007; Martinez-Reyna and Vogel, 2008; Vogel and Mitchell, 2008; Bhandari et al., 2010; Okada et al., 2010). In addition to high above-ground yields, switchgrass can sequester carbon into its extensive root systems, adding to the large positive carbon balance when utilized as a biofuel crop (Liebig et al., 2008; Schmer et al., 2008, 2011).

However, continued improvements in biomass yields, quality, and yield stability will be required to attain a national goal of replacing a portion of petroleum gasoline with liquid fuels derived from lignocellulosic crops by the year 2030 (Perlack et al., 2005; U.S. Department of Energy., 2011). This goal will require sustained high productivity from switchgrass fields. Two important economic drivers for field-scale production of switchgrass occur first during the establishment year (Schmer et al., 2006; Perrin et al., 2008), and later through biotic or abiotic stresses that result in stand and/or yield losses in established fields (Vogel et al., 2002, 2011). What is currently unknown is the extent to which confirmed and as yet unforeseen insect pests can compromise sustainable production of switchgrass and other perennial bioenergy grasses.

Over the course of the last decade, significant investments have been made, principally by the U.S. Department of Energy (DOE), to develop genomic resources for switchgrass and related species. Initial work using a limited number of Sanger-sequenced ESTs (expressed sequence tag; Tobias et al., 2005) identified a number of genes from complementary DNA (cDNA) libraries obtained from different tissues. Subsequently, work undertaken by the DOE-Joint Genomes Institute (DOE-JGI) led to the public release of over 400,000 switchgrass ESTs from a range of tissues. This dataset provided deeper sequencing of transcripts and included several protein families, including peroxidases, that were annotated (Tobias et al., 2008). In 2011, a contig-based early version of the switchgrass genome was released and is available at Phyozome.org (Goodstein et al., 2012); it contains 65,878 gene models arising from the A and B genomes. Based on the number of loci observed in related diploid grasses such as *Sorghum bicolor* with 34,496 loci (Paterson et al., 2009) and *Setaria italica* with 35,471 (Bennetzen et al., 2012; Zhang et al., 2012), it can be anticipated that over 93% of the protein-coding genes have been annotated in the early release of the switchgrass genome. More recently, both next-generation sequencing (NGS) and microarrays have been utilized to probe the transcriptomes of different switchgrass tissues (Palmer et al., 2011; Ersoz et al., 2012; Zhang et al., 2013). The availability of the mostly annotated switchgrass genome along with increasing numbers of NGS transcriptomic datasets presents an opportunity to datamine for expression profiles of genes that could participate in the various aspects of plant development and responses to the environment.

## LIGNIN, ETHANOL, AND PLANT FITNESS

Cell wall composition impacts resistance to pests and pathogens (Santiago and Malvar, 2010; Funnell-Harris et al., 2012), and is a critical component determining biomass quality (Sarath et al., 2008). Plant cell walls consist of three major polymers, namely cellulose, hemicellulose, and lignin (Boerjan et al., 2003). The polysaccharide polymers, cellulose and hemicellulose are the dominant sources of sugars for conversion into liquid fuels in biorefineries. Lignin is an aromatic polymer derived from cytoplasmically synthesized monolignols (Boerjan et al., 2003; Ralph et al., 2006). Monolignols are products of the phenylpropanoid pathway that is also the route for a range of other plant secondary metabolites associated with plant development and defense (Shadle et al., 2003; Zhao and Dixon, 2011). Polymerization of monolignols occurs in the cell wall apoplast after transport across the plasma membrane, catalyzed principally by wall-bound peroxidases and laccases (El Mansouri et al., 1999; Boerjan et al., 2003; Berthet et al., 2011; Cesarino et al., 2013). Lignin content and composition will be driven by the rate, amounts and types of monolignols that are transported to the apoplast. This transport process has not yet been fully elucidated, and previous hypothesis have suggested various mechanisms including passive diffusion, vesicle-mediated transport, facilitated diffusion through channels and active transport via transporters (Fagerstedt et al., 2010). Labeling studies using [^3^H]phenylalanine in lodgepole pine have indicated Golgi-vesicle-mediated transport is unlikely based on the finding that inhibition of protein synthesis decreased the Golgi label while inhibition of phenylpropanoid metabolism did not (Kaneda et al., 2008). More recently, investigations using isolated vesicles from *Arabidopsis* demonstrated that transport of monolignols was an ATP-dependent process for both vacuolar and plasma membrane vesicles (Miao and Liu, 2010). Also, using sodium orthovanadate and a variety of other inhibitors to inhibit ATP-binding cassette (ABC) transporter activity, it was shown that these inhibitors substantially reduced monolignol transport activity, indicating that ABC transporters were likely involved in the transport process. Disruptions to the membrane pH gradient or membrane potential did not exhibit the same degree of transport inhibition (Miao and Liu, 2010). Additionally, the *Arabidopsis* ABC transporter AtABCG29 was recently identified as a *p*-coumaryl alcohol transporter based on several lines of evidence (Alejandro et al., 2012). In this study, yeast strains-expressing AtABCG29 were highly sensitive to *p*-coumaryl alcohol and isolated yeast vesicles from AtABCG29-expressing yeast contained higher levels of *p*-coumaryl alcohol. Also, *abcg29* knockout plant lines exhibited substantially reduced root length in media containing *p*-coumaryl alcohol, and lowered H, G, and S-lignin content based on thioacidolysis yields. RT-qPCR data showed *AtABCG29* was upregulated in WT plants in response to *p*-coumaryl but not sinapyl or coniferyl alcohols (Alejandro et al., 2012). Taken together, this emerging evidence favors the active transport hypothesis and suggests that other monolignols are likely transported to the apoplast via monolignol-specific ABC (or possibly other) transporters that remain to be discovered.

Once transport to the apoplast is complete, monomers are polymerized into developing or new polymers, primarily via radical-coupling mechanisms initiated by multiple classes of apoplastic enzymes including peroxidases, laccases, and oxidases (Boerjan et al., 2003). The basic radical coupling mechanism has long been postulated (Freudenberg, 1959). In general, the polymerization of lignin has appeared to proceed under chemical control rather than the more confining and controlled nature of biochemical control that governs most other plant processes. The proposed mechanism involves dehydrogenation and subsequent polymerization of the radicals; each coupling requires the generation of two radicals and polymerization may proceed through a radical transfer or redox shuttle mechanism since the growing lignin polymer and monolignols such as sinapyl alcohol are not easily oxidized (Boerjan et al., 2003; Liu, 2012).

Within the plant cell walls, lignin forms a physical barrier against the entry of pests and pathogens (Boerjan et al., 2003; Sattler and Funnell-Harris, 2013), acts as an antifeedant for herbivorous insects (Ralph et al., 2006; Deng et al., 2013) and is the major factor impeding the conversion of herbaceous biomass to ethanol (Dien et al., 2009; Fu et al., 2011; Saathoff et al., 2011; Sarath et al., 2011). The negative impact of lignin in the biochemical conversion of herbaceous feedstocks to liquid fuels has led to intense efforts to develop a range of biofuel feedstocks with lowered lignin content (Carroll and Somerville, 2009; Studer et al., 2011; Robbins et al., 2012; Shen et al., 2012). Lowering lignin generally negatively impacts plant fitness, especially in perennial grasses (Casler et al., 2002; Pedersen et al., 2005), although many of molecular and cellular aspects of the biotic interactions of these lower lignin plants remain to be explored.

## CLASS III PEROXIDASES AND LIGNIN BIOGENESIS

Class III peroxidases are ubiquitous plant enzymes that are coded by a large number of related genes within a plant genome (Passardi et al., 2004, 2007; Tobias et al., 2008), and have been implicated in a myriad of plant developmental processes and responses to biotic and abiotic stress (Almagro et al., 2009; Cosio and Dunand, 2009; Gulsen et al., 2010; Linkies et al., 2010; Mika et al., 2010; War et al., 2012). Some peroxidases appear to have a specialized role in lignification. In tobacco, overexpression of a chimeric anionic peroxidase resulted in plants containing higher basal levels of lignin when compared to control plants (Lagrimini, 1991). Wounding of these plants also appeared to result in higher polymerization of phenolic acids, particularly in pith tissue which had much higher levels of peroxidase activity than control plants, although the transgenic plants expressed transgene throughout the plant due to the use of the cauliflower mosaic virus 35S promoter (Lagrimini, 1991). Additional work in tobacco demonstrated that other peroxidases may also be involved in lignification. Antisense gene silencing of the cationic peroxidase TP60 (NtPrx60) in tobacco resulted in several plant lines that exhibited lower lignin content based on thioacidolysis, acetyl bromide and nitrobenzene determinations (Blee et al., 2003). Later work showed some of the generated T1 plants had abnormal phenotypes including discoloration, altered leaf morphologies, and poorly developed xylem in one of the lines (Kavousi et al., 2010). In poplar, a cationic cell wall-bound peroxidase, dubbed CWPO-C, was found to preferentially oxidize sinapyl alcohol monomers as well as sinapyl alcohol polymers (Sasaki et al., 2004). This finding indicated that the suggested radical transfer or redox shuttle mechanisms for lignin polymerization may be unnecessary in at least some circumstances. Other work in tomato demonstrated that overexpression of a tomato basic peroxidase, *tpx1*, resulted in higher cell wall peroxidase activity and higher leaf lignin levels (El Mansouri et al., 1999). A basic peroxidase from *Zinnia elegans* was found to be composed of two isoforms, ZePrx34.70 and ZePrx33.44 that were studied in detail (Gabaldón et al., 2005). Here, the peroxidases were shown to have high affinity for sinapyl alcohol and carried out polymerization of this substrate, suggesting a likely role in polymerization of S-lignin during growth (Gabaldón et al., 2005). Similar results were found for the anionic peroxidases Pxp3, Pxp4, and Pxp5 that were isolated from poplar xylem (Christensen et al., 1998), and several peroxidases from silver birch (*Betula pendula*) and Norway spruce (*Picea abies*) were shown to have activity on monolignol substrates (Marjamaa et al., 2006). In aspen, GUS staining revealed that the anionic peroxidase *prxA3a* was predominately expressed in lignifying stem tissue, particularly xylem (Li et al., 2003). Furthermore, down regulation of this gene using an antisense construct resulted in plants with lower total peroxidase activity and a lowered lignin content that, depending on the transgenic line, approached 20% (Li et al., 2003).

Several peroxidases have been identified in *Arabidopsis* that appear to have roles in lignification. *Arabidopsis* ATP A2 (AtPrx53), a cationic peroxidase, was found to localize to lignified tissues and transgenic plants exhibited differential phloroglucinol staining compared to WT plants; unfortunately, lignin levels were not reported (Østergaard et al., 2000). Modeling based on the ATP A2 crystal structure indicated monolignol substrates could dock in the active site (Østergaard et al., 2000), although class III peroxidases are known to be capable of oxidizing a wide variety of phenolic compounds (Marjamaa et al., 2009). Recently, *Arabidopsis* AtPrx37 was found to be highly expressed in roots as well as flower stems and mature leaves (Pedreira et al., 2011). Overexpression of this gene fused to a GUS reporter gene showed localization in vascular tissue; mutant lines exhibited shorter roots, delayed development and dwarfism, which led to the hypothesis that overexpression of AtPrx37 led to higher cell wall cross-linking (Pedreira et al., 2011). A microarray study in *Arabidopsis* identified eight peroxidases and several laccases with expression profiles that clustered with monolignol synthesis; AtPrx2, AtPrx17, AtPrx37, AtPrx9, and AtPrx30 were peroxidases that were noted to show the strongest co-expression patterns (Ehlting et al., 2005). Gravistimulation was used in one study to alter the mechanical forces acting on stem region of the inflorescence, which was then excised into apical, middle, and basal parts (Yokoyama and Nishitani, 2006). Expression profiling using a microarray showed upregulation of AtPrx42, AtPrx64, and AtPrx71 in basal stem regions compared to middle and apical regions; however, the statistical significance of the expression change was not reported (Yokoyama and Nishitani, 2006). More recently, AtPrx 4, 52, 49, and 72 were suggested to have roles in lignification based on homology to ZePrx and *in silico* characterization of other properties including surface charge, mRNA stability, and amino acid positions (Herrero et al., 2013). Identification of peroxidases that have a primary, or even secondary, role in lignification will likely remain challenging. Despite the fact that a pectate binding site has been identified in an anionic peroxidase from zucchini which suggested possible involvement in lignification (Carpin et al., 2001), further research indicated that the physiological role of the protein involved auxin oxidation in termination of hypocotyl elongation (Cosio and Dunand, 2009). Some characteristic features of syringyl peroxidases have been found. These have included a VSCAD motif compared to a VSCSD motif in G peroxidases as well as the finding that S peroxidases lack helix D’ (Gómez Ros et al., 2007). These changes were postulated to result in conformational changes in the peroxidase active site that allowed sinapyl alcohol to more successfully dock, and thus undergo oxidation (Gómez Ros et al., 2007). In general, basic and neutral peroxidases do not efficiently oxidize sinapyl alcohol owing to steric hindrance of the substrate with the active site (Østergaard et al., 2000). In contrast, acidic peroxidases can oxidize sinapyl alcohol (Sasaki et al., 2004; Gabaldón et al., 2005), indicating that both basic and acidic peroxidases have complementary roles in lignification.

## BIOTIC STRESS, ROS, AND PEROXIDASES

An essential function for class III peroxidases is to protect the cellular membranes against oxidative damage. More specifically, class III secreted peroxidases are players in both reactive oxygen species (ROS) removal and ROS generation (Passardi et al., 2005). Although high levels of ROS are deadly, sub-lethal levels of ROS can serve as signals, prompting cells to prepare for sustained oxidative stress (Miller et al., 2009; Torres, 2010). Due to the potential exposures of deadly ROS, higher plants have evolved enzymes to detoxify these molecules (Mittler et al., 1999). Catalases, peroxidases and superoxide dismutase have all been documented as ROS scavengers in plants stressed by insects and pathogens (Felton et al., 1994b; Heng-Moss et al., 2004; Franzen et al., 2007; Kusnierczyk et al., 2008).

Plants have also evolved complex signaling networks intended to detect specific pathogens in order to trigger the appropriate defense responses. A growing body of evidence suggests that plants have evolved intricate mechanisms to exert control over pathogen induced defense pathways. The hypersensitive response (HR) is a complex early defense response that causes necrosis and cell death that can restrict the growth and spread of a pathogen. This interaction leads to a change in the membrane potential and ion permeability of the host cell plasma membrane resulting in localized cell death (Heath, 2000). One of the first biological responses of the HR is an oxidative burst which includes the generation of ROS including hydroxyl radicals (-OH), nitric oxide (NO), hydrogen peroxide (H_2_O_2_), and superoxide (Apostol et al., 1989). Left unchecked, these ROS may cause protein, lipid, and nucleic acid damage (Roldán-Arjona and Ariza, 2009; Sharma et al., 2012).

Peroxidase activity and/or peroxidase gene expression has been shown to be induced by many types of pathogens including fungi (Sasaki et al., 2004; Wang et al., 2013), bacteria (Young et al., 1995; Lavania et al., 2006), viruses (Lagrimini and Rothstein, 1987; Hiraga et al., 2000; Díaz-Vivancos et al., 2006; Babu et al., 2008) and viroids (Vera et al., 1993). These studies reinforce the hypothesis that class III peroxidases have important roles in plant defense and can serve as markers of plant responses to biotic stressors.

## PEROXIDASES AND DEFENSE AGAINST INSECTS

Insect infestation and herbivory has often been linked to changes in cellular ROS and peroxidase activity (Hiraga et al., 2001; Ni et al., 2001; Kawano, 2003; Heng-Moss et al., 2004; Passardi et al., 2005; Torres, 2010; O’Brien et al., 2012; War et al., 2012). Plant peroxidase levels in response to hemipterans have been particularly well-studied (Hildebrand et al., 1986; Felton et al., 1994a,b; Stout et al., 1999; Ni et al., 2001; Heng-Moss et al., 2004). It has been observed that peroxidase levels increase following chinch bug and aphid feeding in tolerant buffalograsses, sorghum, and barley (Heng-Moss et al., 2004; Franzen et al., 2007; Gulsen et al., 2007, 2010). Recently, studies have shown that in wheat, rice, switchgrass and tomato class III peroxidases transcripts were upregulated in response to insect herbivory (Dowd and Johnson, 2009; Liu et al., 2010; Suzuki et al., 2012; War et al., 2012).

It is likely that the abundance of several class III peroxidases identified in switchgrass (see below) will also play significant roles in tolerances against insects. Previous global analysis in rice and wheat challenged with Hessian fly attack identified 34 class III peroxidases that were upregulated in resistant plants versus 22 peroxidases in susceptible plants (Liu et al., 2010). In *Arabidopsis*, two peroxidases, At5g64120 (AtPrx71) and At5g05340 (AtPrx52), were found to be induced by *Pieris brassicae* eggs were also induced by *Pieris rapae* herbivory (Little et al., 2007). For a better understanding of the roles of individual peroxidases in switchgrass against hemipterans, experiments using both resistant and susceptible cultivars challenged with a variety of these potential pests should be conducted. It is likely that class III peroxidases will display similar roles in switchgrass when challenged with hemipterans as well. Currently, roles of most of these proteins in plant defense or resistance in switchgrass are not known, but appear to be important areas of future research.

## ENERGY CROP–INSECT INTERACTIONS

One of our long-term research goals is to develop a molecular understanding of switchgrass responses to hemipterans, utilizing a selection of tetraploid switchgrasses. The incidence of arthropod pests is likely to increase in switchgrass systems due to an anticipated shift to monoculture-based biomass production systems (Vogel et al., 2011). Additionally, due to plant-breeding efforts to reduce traits that interfere with biofuel processing, some plant defense mechanisms may be negatively impacted (Nabity et al., 2012). While switchgrass may be one of the better studied warm-season native grasses, most research has focused on agronomic qualities and disease issues. Few studies have examined the arthropod communities associated with switchgrass (Boerner and Harris, 1991; Gottwald and Adam, 1998; Kindler and Dalrymple, 1999; McIntyre and Thompson, 2003; Schaeffer et al., 2011). This basic information is required to identify and define the organisms that may cause reduced yields. Although much uncertainty exists in predicting which insects will be the key pests of this new market-use of switchgrass, recent research has provided clear evidence that biomass crops are susceptible to a number of key pests of other important crop plants (Prasifka and Gray, 2012). In switchgrass, potential pests have included stem-boring insects (Prasifka et al., 2010, 2011b), defoliators (Prasifka et al., 2009, 2011a) and piercing-sucking insects (Schaeffer et al., 2011; Burd et al., 2012). Some of these insects have broad host ranges with multiple biotypes (Prasifka et al., 2009), while others are apparently very specific to switchgrass (Adamski et al., 2010; Prasifka et al., 2010).

Importantly, our development of switchgrass as a biofuel feedstock is, in part, a response to global climate change (Energy Independence and Security Act of 2007, 42 U.S.C. § 17001), to which some desirable insect species (Pelini et al., 2010) will not adapt and some important pest species will overcome (Davis et al., 2006). Aphids (winged aphids in particular) transmit viruses with their mouthparts and are the most predominant vector for plant viruses (Hull, 2001). Depending on the virus, they can remain on the aphid’s mouthparts (specifically, their stylets) or circulate throughout the vector (some viruses replicate within the aphid) prior to transmission. Aphids are well-known to sample plant tissues (i.e., probing behavior) with their stylets to determine host acceptability. Additionally, some aphid species alternate between plant species as a function of their seasonal life cycle. This probing and host-alternation behavior of aphids is highly conducive to the introduction of new plant–virus relationships. For the above reasons, we have begun to explore the potential for targeting peroxidases in switchgrass genotypes for focused plant-breeding efforts.

## PHYLOGENETIC RELATIONSHIPS OF SWITCHGRASS PEROXIDASES

Peroxidases are grouped into one of two superfamilies. One superfamily, the peroxidase–cyclooxygenase superfamily, generally consists of animal peroxidases that are structurally unrelated and important in the innate immune system (Söderhäll, 1999). The other superfamily, the peroxidase–catalase superfamily, includes plant, fungal, and bacterial peroxidases. The catalase–peroxidase superfamily is further divided into three distantly related structural classes (Welinder, 1992). Plant peroxidases fall into the first and third classes and are heme-containing enzymes that play key roles in important biological process such as biosynthesis of lignin, degradation pathways and host-defense mechanisms. Class I peroxidases are intracellular peroxidases without signal peptides, calcium ions, or disulfide bridges. They show moderate substrate specificity of ascorbic acid and are located in the chloroplasts, mitochondria, peroxisomes, and the cytosol (Almagro et al., 2009). Extracellular secretory fungal peroxidases comprise class II peroxidases (Reddy, 1993) that include lignin-modifying peroxidases and manganese peroxidases. Class III peroxidases are glycoproteins that are located in vacuoles and cell walls and further divided into eight distinct groups based on sequence (Passardi et al., 2004). These peroxidases are involved in cell elongation, cell wall construction, and responses to various abiotic stresses and biotic plant pathogens (Intapruk et al., 1994; Klotz et al., 1998; González-Rábade et al., 2012).

In the PeroxiBase database (), a total of 8,695 peroxidases have been collected. Of those, 5,430 (approximately 61%) are class III peroxidases which have been identified (March, 2013) from multiple plants species. For example, the genomes of *Brachypodium distachyon*, *Arabidopsis thaliana*, and *Oryza sativa* appear to code for 143, 73, and 155 class III peroxidases, respectively (Welinder et al., 1996; Passardi et al., 2004; Vogel et al., 2010). Comparisons of the peroxidase families between rice and *Arabidopsis* led to a better understanding of the evolution of monocots and dicots that diverged from a common ancestor 150 million years ago (Wikström et al., 2001). Monocotyledon peroxidases differ slightly in intron/exon size and structure from Eudicotyledons, but the majority of class III peroxidases are highly conserved.

**FIGURE 1:**
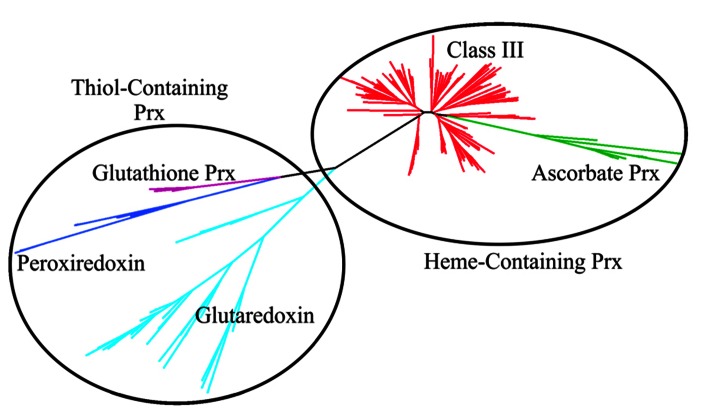
**Phylogenetic clustering of heme- and thiol peroxidases (Prx) present in the switchgrass genome.** Heme peroxidases (oval) consist of the class I ascorbate peroxidases (green), and class III peroxidases (red). Thiol peroxidases (circle) consist of the glutathione peroxidases (purple), peroxiredoxin (blue) and glutaredoxin (cyan).

**FIGURE 2:**
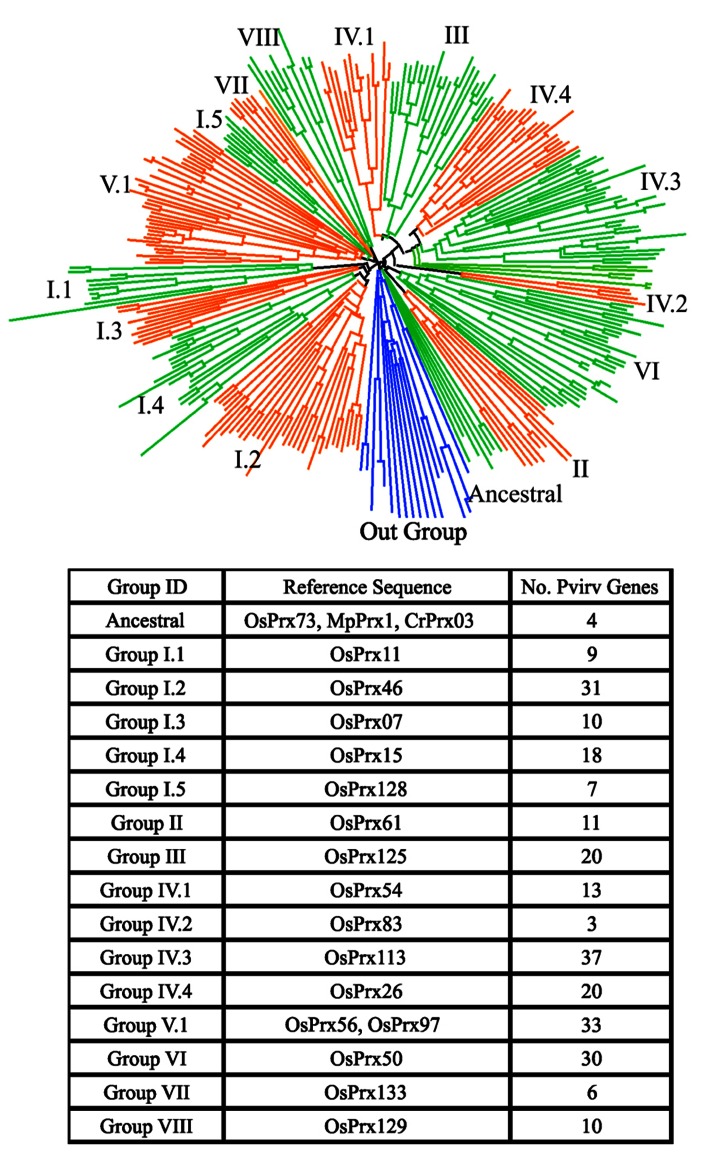
**Phylogenetic relationships and numbers within each evolutionary clade, as defined for rice, for switchgrass class III peroxidases.** The distribution of peroxidases relative to the out-group sequences (blue lines) are shown in the circular phylogram. The rice and ancient peroxidase protein sequences used as representatives for these analyses, and the numbers of switchgrass members in each clade are indicated. The peroxidase sequences include loci from both the A and B genomes.

In switchgrass, the preliminary classification of sequences obtained from a cDNA library showed class III peroxidases were extremely well-represented, with approximately 400 EST’s identified (Tobias et al., 2008). Using representative proteins belonging to different peroxidase groups in rice, all putative matches were identified in the switchgrass genome using the Blastp algorithm (Altschul et al., 1990, 1997) and an e-value threshold of 1 × 10^-15^. Putative matches and representative rice protein sequences were aligned using FastTree (Price et al., 2009) and visualized using Dendroscope version 2 (Huson et al., 2007). Two major peroxidase subfamilies, the heme and thiol peroxidases were separated. The thiol peroxidases were further subdivided into glutaredoxins, peroxiredoxins and glutathione peroxidases. The heme-containing switchgrass peroxidases were separated into class III and ascorbate (Class I) peroxidases (**Figure 1**). The class III peroxidases were further analyzed by reclustering and seeded with rice proteins belonging to different evolutionary groups of class III peroxidases (Passardi et al., 2004). Based on these analyses, the ancestral switchgrass peroxidase genes were identified as Pavirv00010935m, Pavirv00050429m, Pavirv00055443m, and Pavirv00060522m. The monocot-specific clade Group V.1 contained 33 members (**Figure 2**).

A comparison of switchgrass peroxidases with two rice class III peroxidases induced during gall midge attack (Liu et al., 2010) found the most similar switchgrass gene for Os07g0677200 was Pavirv00031572m and for Os06g0547400 was Pavirv00059991m. In rice, these two genes were significantly elevated in expression at 12 h post attack and eventually decreased in expression after 72 h post. It is possible that these two class III peroxidases in switchgrass may be potential sources for ROS production and defense during an insect attack as well. Two other class III peroxidases associated with insect defense from *Arabidopsis* (Cosio and Dunand, 2009) share similarity with switchgrass peroxidases. The *Arabidopsis* protein PRX52 (AT5G05340) shares similarities with Parvirv00018711m and PRX71 (At5g64120) is homologous to Pavirv00006707m. These findings shed light on possible peroxidase targets to evaluate during insect attack.

## GLOBAL ANALYSIS OF PEROXIDASE TRANSCRIPTS IN SWITCHGRASS TISSUES

**FIGURE 3:**
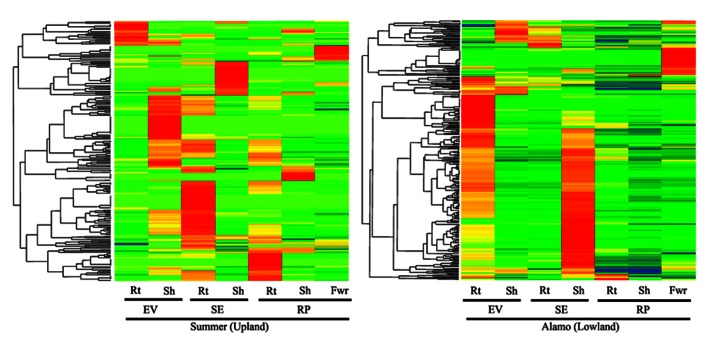
**One-way clustering color map for class III peroxidase expression profiles (z-scores) for publically available NGS datasets for cultivars Summer and Alamo.** Stages of plant development are early vegetative (EV), stem elongation (SE), and reproductive (RP) are as described in these datasets. Roots = Rt; Shoot = Sh; Flowers = Fwr. Red indicates high abundance, yellow is intermediate and green and blue are low or negligible abundance. The appropriate SRA identification numbers for these individual NGS files are shown in**Table A1** in Appendix.

To probe the profiles of class III peroxidase expression in different switchgrass populations and plant organs, we queried transcriptomic datasets obtained using NGS platforms (Roche 454 Life Sciences instrument; **Table A1** in Appendix). Publically available NGS datasets from two contrasting ecotypes of switchgrass (lowland cv. Alamo and upland cv. Summer) were mined to obtain class III peroxidase expression profiles in different tissues (**Figure 3**). There appears to be significant differences in the profiles of class III peroxidase genes expressed in the different tissues based on plant developmental stage and ecotypes. Clusters of class III peroxidases were strongly represented in roots and shoots harvested from plants at early vegetative (EV), stem elongation (SE), and reproductive (RP). At each of these stages in Summer, there appeared to be strong transcriptional control of the expression of specific sets of class III peroxidase genes. As an example, many peroxidases were upregulated in roots during the SE stage of harvest (**Figure 3**), possibly related to greater root growth at this stage of plant development. Transcripts for many of these genes were less abundant at the RP stage, although expression of a different cluster of genes in the roots of Summer plants was apparent. In the shoots of Summer plants, greatest abundance in peroxidase transcripts apparently occurred during stages of active tiller elongation (EV and SE; **Figure 3**), suggestive of roles in cell wall formation and active tiller growth processes. There was enrichment in transcripts for a small number of potentially organ-specific peroxidases in flowers. In the lowland cultivar Alamo, there appeared to be notable differences in class III peroxidase gene expression patterns relative to Summer plants. Maximal transcript abundances for roots were observed at the EV stage of growth, and a majority of these transcripts were found in lower abundances at later harvest dates. However, the physiological significance of these initial observations is unclear. For Alamo shoots, a similar pattern to those described for roots were seen, except that highest apparent transcript abundance was seen at the SE stage of growth (**Figure 3**). A small cluster of peroxidases were upregulated in Alamo flowers, similar to what was observed from these NGS datasets for Summer flowers (**Figure 3**). Flower-specific peroxidases have been reported in the literature (Cosio and Dunand, 2009; Beltramo et al., 2012).

**FIGURE 4:**
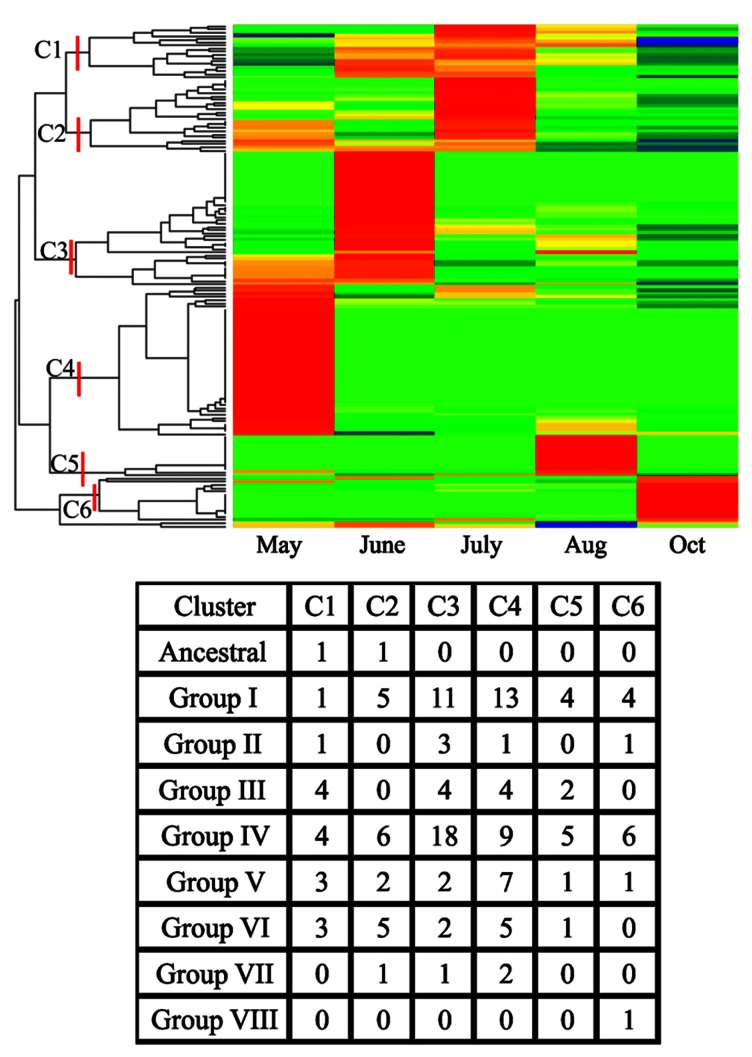
** for crowns and rhizomes obtained from field-grown Summer plants at the Agricultural Research and Development Center fields of the University of Nebraska.** Harvest months are shown and coincided approximately with green-up (May), late vegetative stage (June), flowering (July), hard-seed stage (Aug), and aerial senescence after a killing frost (Oct). Red indicates high abundance, yellow is intermediate and green and blue are low or negligible abundance. The appropriate SRA identification numbers for these individual NGS files are shown in**Table A1** in Appendix. The six clusters of the most abundant transcripts from each harvest date are shown (C1–C6) along with the numbers of individual peroxidases as assigned to an evolutionary group.

An analysis of crown and rhizome transcriptomes for expression profiles of class III peroxidases obtained from field-grown plants is shown in **Figure 4**. Tissues were harvested from plants over the course of a growing season as described earlier (Palmer et al., 2011). Since these tissues are critical for perenniality of the plants, knowledge of the molecular mechanisms that might impact perenniality will be useful both from a biological and breeding perspective. Below ground herbivory and attack from other pathogens can result in reduced shoot biomass, negatively impact plant survival and overall system sustainability (for example corn root worm, nematodes, etc.). Peroxidases can serve as effective markers for plant stress (reviewed above) and understanding expression profiles will provide insights into the cellular state of these tissues.

As observed for other switchgrass tissues (see **Figure 3**), there were clusters of peroxidases overexpressed at specific harvest dates (**Figure 4**). These field harvest dates coincided approximately to spring emergence (green-up; May), late vegetative (June), flowering (July), late seed set (August), and senescence of aerial tissues after a killing frost (October). The largest number of strongly upregulated peroxidases appeared to occur in May and June, coinciding with a time of rapid vegetative growth, somewhat similar to the patterns seen in the Alamo datasets. The total numbers of peroxidase transcripts exhibiting greater expression declined at the last three harvest dates, with relatively few genes overexpressed at the August and October harvests (**Figure 4**). Some of these genes appear to be specific for a given harvest date, and are probably reflective of the developmental stage of the plants. These highly expressed genes were separable into six clusters (C1–C6, **Figure 4**). Peroxidase distribution in these clusters based on their phylogenetic classification (**Figure 2**) suggested members of different groups became active at various times throughout the growing season. In May, highly expressed peroxidases clustered mainly into C4, with some upregulation in C3 and C2 apparent. In C4, the highest numbers of peroxidases came from Group I and Group IV, with lower amounts in other groups. In June, most peroxidase upregulation clustered into C3, with some upregulation in C1. In the larger C3 cluster, upregulated peroxidases were found again in Groups I and IV, with the latter containing double the number of upregulated members when compared to May. In July, upregulated peroxidases clustered into two groups (C1 and C2); cluster C1 had highly expressed members at approximately equal levels from Groups III, IV, V, and VI while cluster C2 contained a slightly different breakdown: Group I, IV, and VI contained most of the apparently upregulated peroxidases. August and October peroxidase expression mainly clustered into C5 and C6, respectively, which showed upregulation primarily in Groups I and IV in both cases. Based on these data, it is apparent that different peroxidases become active in switchgrass crown and rhizome tissue as the plant transitions from active growth through flowering and into dormancy, and that the study of class III peroxidases is likely to yield significant insights into switchgrass developmental processes and the interactions of the plant with biotic and abiotic stress.

## CONCLUSION

Sustainable production of switchgrass and other bioenergy grasses will require effective management against biotic stressors. The need to raise these crops on marginal land with lowered inputs will necessitate developing cultivars with enhanced tolerance to a range of biotic and abiotic stresses. We are only now beginning to probe the genotypic diversity that exists in switchgrass populations to potential insect pests. Identification of potential insect pests and detailed characterization of the plant–insect interaction will better enable us to address emergent insect pests in switchgrass production fields. Additionally, it is unclear how manipulation of plants for quality traits (for example lower/higher lignin) will affect plant resistance to insect herbivory and other endogenous mechanisms that confer resistance. However, based on extensive scientific literature, it can be safely predicted that the class III peroxidases are going to play a key role in the defensive mechanisms of switchgrass plants to insect herbivory, specifically to insects containing piercing-sucking mouthparts. The combination of genomic resources and improved phenotyping methods are likely to help decipher these molecular circuits, and provide guidance for the continued improvements of switchgrass as a bioenergy feedstock.

## Conflict of Interest Statement

The authors declare that the research was conducted in the absence of any commercial or financial relationships that could be construed as a potential conflict of interest.

## Appendix

**Table A1:** Switchgrass NGS datasets.

Cultivar	Time	Tissue	# Reads	SRA
Summer	May	Crown	445,432	SRX257007
Summer	June	Crown	379,264	SRX257030
Summer	July	Crown	317,557	SRX257031
Summer	August	Crown	930,114	SRX102934
Summer	October	Crown	389,555	SRX257032
Summer	Early vegetative stage	Root	265,190	SRX026147
Summer	Early vegetative stage	Shoot	211,124	SRX026148
Summer	Stem elongation stage	Root	187,893	SRX026150
Summer	Stem elongation stage	Shoot	210,071	SRX026149
Summer	Reproductive stage	Root	240,166	SRX026153c
Summer	Reproductive stage	Shoot	228,101	SRX026151c
Summer	Reproductive stage	Flower	240,696	SRX026155c
Alamo	Early vegetative stage	Root	1,317,713	SRX057831
Alamo	Early vegetative stage	Shoot	1,298,485	SRX057830
Alamo	Stem elongation stage	Root	1,113,868	SRX057829
Alamo	Stem elongation stage	Shoot	1,407,916	SRX057828
Alamo	Reproductive stage	Root	1,037,727	SRX057826
Alamo	Reproductive stage	Shoot	557,570	SRX057827
Alamo	Reproductive stage	Flower	1,143,746	SRX057834
